# Emergency teleradiological activity is an epidemiological estimator and predictor of the covid-19 pandemic in mainland France

**DOI:** 10.1186/s13244-021-01040-3

**Published:** 2021-07-22

**Authors:** Amandine Crombé, Jean-Christophe Lecomte, Nathan Banaste, Karim Tazarourte, Mylène Seux, Hubert Nivet, Vivien Thomson, Guillaume Gorincour

**Affiliations:** 1 Imadis Teleradiology, Lyon, Bordeaux, Marseille, France; 2 University of Bordeaux, Bordeaux, France; 3 Centre Hospitalier de Saintonge, Saintes, France; 4 Centre Aquitain D’Imagerie, Bordeaux, France; 5 Department of Radiology, Hôpital Nord-Ouest, Villefranche-sur-Saône, France; 6 Emergency Department, CHU Edouard Herriot, Hospices Civils de Lyon, Lyon, France; 7 INSERM 1290 RESHAPE, University of Lyon 1, Lyon, France; 8 Ramsay Générale de Santé, Clinique de la Sauvegarde, Lyon, France; 9 ELSAN, Clinique Bouchard, Marseille, France

**Keywords:** Coronavirus infections, Teleradiology, Public health, Forecasting

## Abstract

**Background:**

COVID-19 pandemic highlighted the need for real-time monitoring of diseases evolution to rapidly adapt restrictive measures. This prospective multicentric study aimed at investigating radiological markers of COVID-19-related emergency activity as global estimators of pandemic evolution in France. We incorporated two sources of data from March to November 2020: an open-source epidemiological dataset, collecting daily hospitalisations, intensive care unit admissions, hospital deaths and discharges, and a teleradiology dataset corresponding to the weekly number of CT-scans performed in 65 emergency centres and interpreted remotely. CT-scans specifically requested for COVID-19 suspicion were monitored. Teleradiological and epidemiological time series were aligned. Their relationships were estimated through a cross-correlation function, and their extremes and breakpoints were compared. Dynamic linear models were trained to forecast the weekly hospitalisations based on teleradiological activity predictors.

**Results:**

A total of 100,018 CT-scans were included over 36 weeks, and 19,133 (19%) performed within the COVID-19 workflow. Concomitantly, 227,677 hospitalisations were reported. Teleradiological and epidemiological time series were almost perfectly superimposed (cross-correlation coefficients at lag 0: 0.90–0.92). Maximal number of COVID-19 CT-scans was reached the week of 2020-03-23 (1 086 CT-scans), 1 week before the highest hospitalisations (23,542 patients). The best valid forecasting model combined the number of COVID-19 CT-scans and the number of hospitalisations during the prior two weeks and provided the lowest mean absolute percentage (5.09%, testing period: 2020-11-02 to 2020-11-29).

**Conclusion:**

Monitoring COVID-19 CT-scan activity in emergencies accurately and instantly predicts hospitalisations and helps adjust medical resources, paving the way for complementary public health indicators.

**Supplementary Information:**

The online version contains supplementary material available at 10.1186/s13244-021-01040-3.

## Key points

- Teleradiological activity in emergency centres is correlated with the number of hospitalisations due to COVID-19 at a nationwide scale in mainland France.
- Monitoring teleradiological activity in emergency centres could improve the detection of COVID-19 resurgence.
- Teleradiological indicators could help forecasting the evolution of the pandemic.

## Background

Coronavirus disease 2019 (COVID-19) has rapidly spread worldwide since its identification in China in December 2019, putting populations, health services and economies under high pressure. To adapt restrictive social and economic measures, governments and national public health agencies require reliable markers to instantly monitor the pandemic.

In France, this mission was assigned to *Santé Publique France* (SPF), which collects daily data from various sources, such as numbers of teleconsultations, laboratory tests, hospitalisations, visits in emergency services, intensive care unit (ICU) admissions, deaths at home or hospital and cases in nursing homes [1].

Retrieving these data from each sub-territory for aggregation at the national level requires considerable resources because of the lack of interoperability and homogenisation of information and technology frameworks in healthcare systems [2, 3].

Among these indicators, the incidence of hospitalisations is particularly scrutinised, as it is closely followed by the incidence of ICU admissions and deaths. Decisions to put countries under lockdown were mostly guided by these figures to avoid overwhelming health services and having to choose which patients to treat.

Teleradiology is a recent human and technological solution that uses homogeneous and interoperable information and technology tools to remotely interpret medical images coming from various healthcare centres. Our structure is dedicated to emergency imaging with a network of partner emergency centres distributed across mainland France. Images are centralised in three interpretation centres where teams of radiologists use the same tools to interpret images and report findings to emergency physicians. Moreover, resource organisation and structured processes, particularly chest CT, provide high diagnostic accuracy with strong inter-observer agreement between on-call teleradiologists with varying degrees of experience and senior radiologists [4]. Indeed, thanks to our structured organisation, we already showed that teleradiology enabled us to monitor the impact of the COVID-19 pandemic management on emergency activities, showing a global decrease in the population's use of care during the first lockdown in France [5]. As we observed similarities between the teleradiology emergency time series and the hospital time series published by SPF, the purpose of this study was to investigate whether structured emergency teleradiology activity could help public health agencies monitor COVID-19 pandemic on a nationwide scale [6].

## Methods

### Study design and epidemiological data

**Fig. 1:**
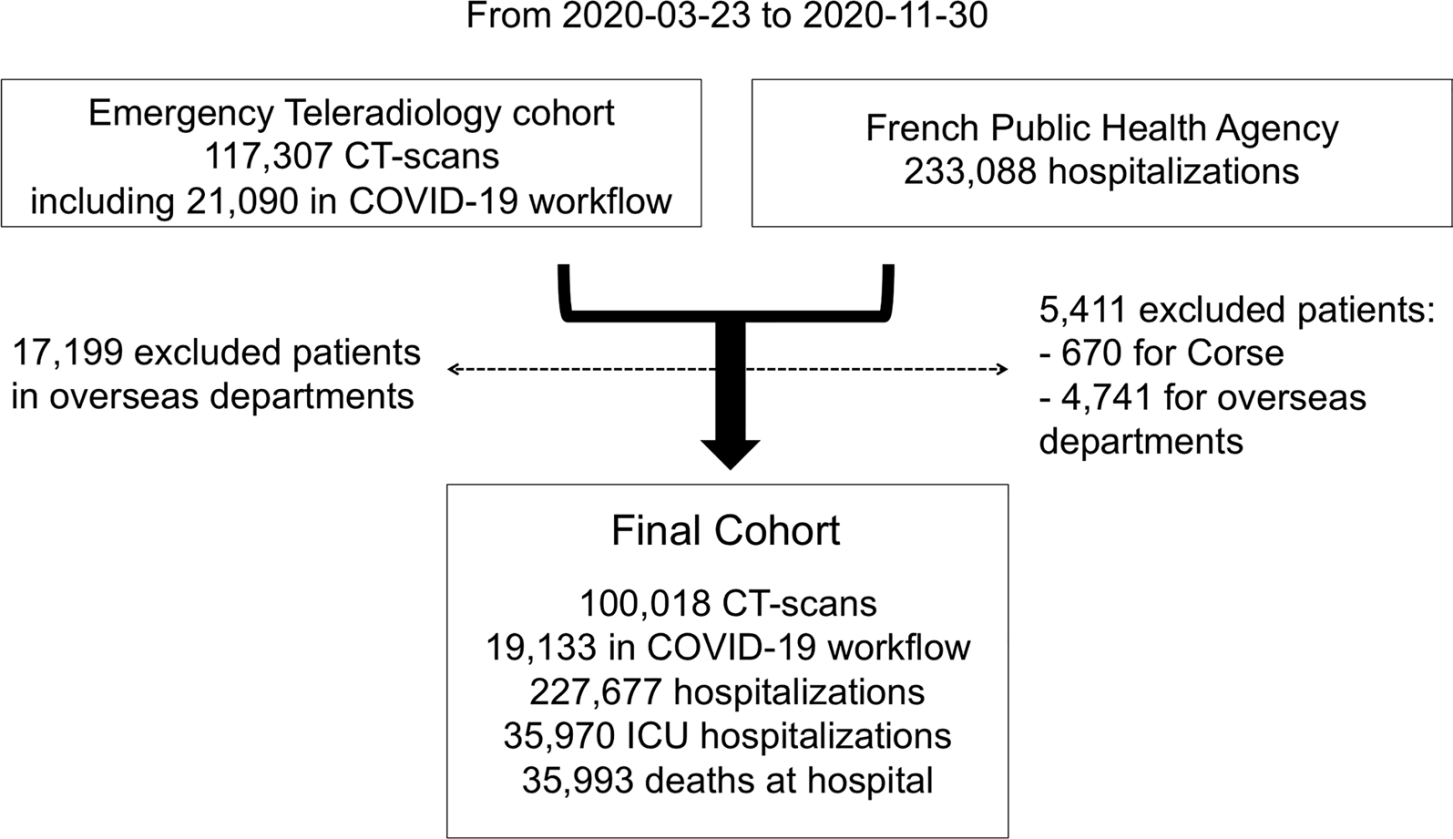
Study flow chart. Abbreviation: ICU: intensive care units. The data from the French public health agency (Santé Publique France) can be found at https://www.data.gouv.fr/fr/datasets/donnees-hospitalieres-relatives-a-lepidemie-de-covid-19/)

This prospective multicentric observational study was approved by the French radiological ethics review board (N° CRM-2012-120). The flow chart is displayed in Fig. 1. All teleradiology patients gave written informed consent for the reuse of anonymised data.

The epidemiological dataset was retrieved from *data.gouv.fr*, an open-source platform storing public datasets promoted by the French government [7]. We used the ‘hospital dataset’, which contains the daily incidences of ICU admissions and standard hospitalisations, deaths in hospital and discharges to home per territorial area. We excluded French overseas departments. We computed the weekly number of hospitalisations, ICU admissions and deaths from Monday to Sunday and indexed this number on the first day of the week. Since the dataset began on Thursday 2020-03-19, we windowed the study from the following Monday (2020-03-23) to the last Sunday of November (2020-11-29).

### Emergency teleradiological data collection

Our teleradiology structure is dedicated to emergency imaging (radiographs, CT-scans and MRI), 24 h a day, 7 days a week, with its main activity during weekends and on-call duty periods (6 pm to 8.30 am). During the study period, 52–65 emergency partner centres distributed across mainland France performed CT-scans that were reviewed by teams of teleradiologists gathered in 3 teleradiology centres in Bordeaux, Lyon and Marseille. Through our dedicated Radiological Information System (ITIS, DeepLink Medical, Lyon, France), a COVID-19 workflow has been implemented since 2020-03-09 that combines a standardised CT request form for requesting physicians, standardised acquisition protocols, structured CT reports and systematic review by a senior radiologist. Regarding the CT-scan acquisitions, chest CT examinations were performed by using 16- or 64-detector row CT scanners with a standardised non-contrast enhanced COVID-19 chest CT protocol for all partner hospitals. The slice thickness ranged from 1 to 1.25 for the lung kernel, and from 2 to 2.5 mm for the mediastinal kernel. If pulmonary embolism was suspected, CT pulmonary angiographic protocol with bolus tracking intravenous iodine contrast agent administration at a rate of 3–4 mL/s was used instead.

The inclusion criteria for the teleradiological cohort were: request for a chest CT-scan in the COVID-19 workflow because of suspicion, diagnosis or clinical aggravation by an emergency physician; achievement of this chest CT-scan and availability of the report. None of the examination were scheduled and/or performed for late stage of the disease or post-COVID-19 symptoms. All requests had to follow the guidelines from the French National High Authority for Health (‘*Haute Autorité de Santé*”) [8].

This process enabled us to prospectively collect the weekly number of CT-scans performed for COVID-19 suspicion, diagnosis or clinical aggravation and to calculate the percentage of activity of this workflow (defined as this number divided by the total number of CT-scans and MRIs reported the same week in our structure). As of 2020-07-06, the number of CT-scans with conclusions stating compatibility with/strongly suggestive of COVID-19 diagnosis (in agreement with the French Society of Radiology standardised report) was prospectively collected [9]. Thus, the percentage of chest CT-scans with compatible/strongly suggestive findings among the chest CT-scans performed in the COVID-19 workflow was calculated for each week following 2020-07-06.

### Statistical analyses

Statistical analyses were performed from 2020-03-23 to 2020-11-29 with R (version 3.5.3, R Foundation for Statistical Computing). A *p* value < 0.05 was deemed significant.

#### Correlations between time series

The cross-correlation function (CCF) was used to identify linear relationships between lagged values of the teleradiological time series and the weekly number of hospitalisations [10]. For each *k* lag between two time series *x*(*t*) and *y*(*t*) composed of *n* observations with SD_*x*(*t*)_ and SD_*y*(*t*)_ as their standard deviations, respectively, the following coefficient was calculated:

$$r\left( {x + k,y} \right) = \left[ {\frac{1}{n}~ \times ~\mathop \sum \limits_{{t = 1}}^{{n - k}} \left( {~y\left( t \right) - ~\bar{y}} \right) \times \left( {x\left( {t + k} \right) - \bar{x}} \right)} \right]~ \times \frac{1}{{\sqrt {{\text{SD}}_{{x\left( t \right)}} \times {\text{SD}}_{{y\left( t \right)}} } }}$$

A significant correlation was retained if it was above or below $- \frac{1}{n} \pm 2/\sqrt n$.

#### Extreme values and structural changes

We reported the minimal and maximal values of each variable and the corresponding weeks when these values were reached. The breakpoints function (“strucchange” package) was applied to investigate structural changes and breaks during the inter-wave period that could have helped identify a 2nd wave primer [11]. This function uses a dynamic programming algorithm and identifies the optimal number of breakpoints in a time series (considered piecewise linear models) that minimises the residual sum of squares as well as the number of parameters in the model.

*Forecasting*. The dataset was divided into one training set (from 2020-03-23 to 2020-11-02) and one validation set (from 2020-11-03 to 2020-11-29). By using the auto.arima function (“forecast” package), we developed dynamic regression models to predict the number of hospitalisations in a given week (*H*(*t*)) in the training set depending on the following possible predictors: number of CT-scans performed the same week (CT(*t*)), one week before (CT(*t* − 1)), and two weeks before (CT(*t* − 2)); number of hospitalisations one week before (H(*t* − 1)), and two weeks before ((*H*(*t* − 2)); and occurrence of national lockdown (Ld(*t*)) [12, 13]. In dynamic regressions, a time series *y*(*t*) is expressed as a linear function of k predictor time series plus an error term (*η*(*t*)) that may contain auto-correlation with its prior values (*η*(*t* − 1), *η*(*t* − 2), …etc.) and is assumed to follow an auto-regressive integrated moving average (ARIMA) model with the *p* and *q* parameters corresponding to the order of the auto-regressive part and the order of the moving average part, respectively, as follows [14]:

$$\begin{aligned} y\left( t \right) & = ~\beta _{O} + \left( {\mathop \sum \limits_{{i = 0}}^{k} \beta _{i} ~ \times ~x_{i} \left( t \right)} \right) + ~~\eta \left( t \right) \\ \eta \left( t \right) & = ~\left( {\mathop \sum \limits_{{i = 1}}^{p} \varphi _{i} \times \eta \left( {t - i} \right)\varphi _{1} } \right) + ~\varepsilon \left( t \right) + \left( {\mathop \sum \limits_{{i = 1}}^{q} \theta _{q} \times ~\varepsilon \left( {t - q} \right)} \right)~ \\ \end{aligned}$$

where *x*_*k*_ are the predictors, *β*_*k*_ are their coefficients, *β*_0_ is the intercept, *ε* is white noise (following a Gaussian law), and *φ* and *θ* are the key parameters of the auto-regressive part and moving average part of the model, respectively. Herein, no integrated part was needed. We evaluated the quality of the fitted models with the corrected Akaike information criterion (AICC), which penalises models that incorporate large numbers of predictors. We verified the validity of the models using the Ljung–Box Q test, which assesses whether the residuals of the model behave like a white noise series [15]. Models with a *p* value < 0.05 were considered to have a lack of fit. Finally, the performance of the models in the training and testing sets was evaluated with the mean average percentage (MAPE), which is a measure of prediction accuracy for forecast models, defined as:

$${\text{MAPE}} = ~\frac{1}{N}~ \times \mathop \sum \limits_{{i = 1}}^{N} \frac{{\left| {H\left( t \right) - ~\widehat{{H\left( t \right)}}} \right|}}{{H\left( t \right)}}~ \times ~100$$

where *N* is the number of observations and $\widehat{{H\left( t \right)}}$ is the fitted value of the number of hospitalisations during week *t*. The smallest value of the MAPE in the test set indicated the best model [13, 16].

## Results

### Visualisation of the epidemiological (nationwide data) and teleradiological time series (teleradiological data)

**Fig. 2:**
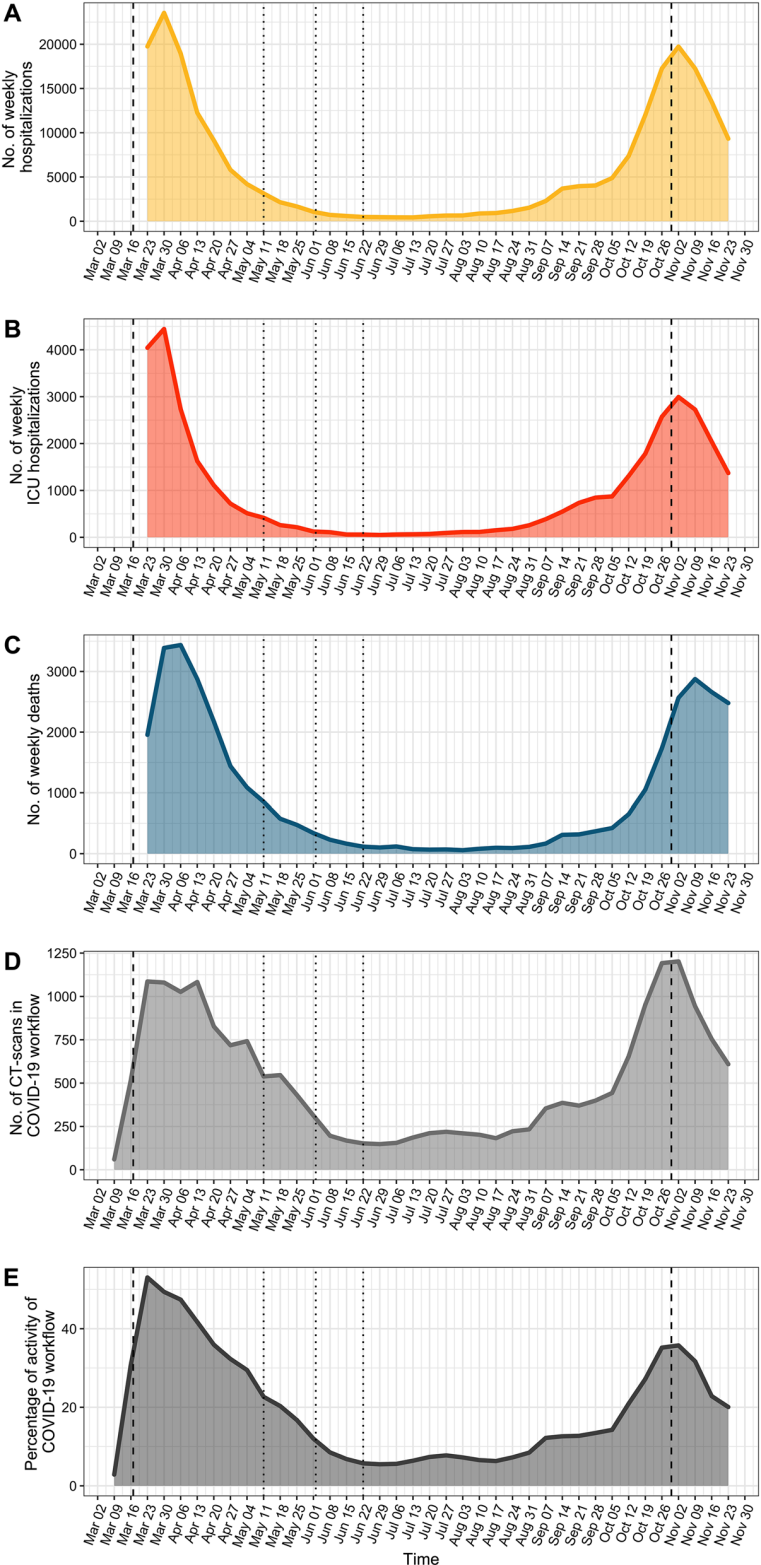
French weekly temporal evolution of: (**a**) new COVID-19-related hospitalisations, (**b**) new COVID-19-related intensive care unit (ICU) admissions, (**c**) new COVID-19-related hospital deaths, (**d**) CT-scans performed in the COVID-19 emergency teleradiological workflow and (**e**) percentage of activity of the COVID-19 workflow (i.e. number of CT-scans related to COVID-19 over the total number of CT-scans). The dashed lines correspond to the dates of 1st and 2nd lockdown beginnings (2020-03-14 and 2020-10-30). The dotted lines correspond to the dates of post-lockdown phases (2020-05-11, 2020-06-02 and 2020-06-22). *Note*: Source of A, B, C curves is epidemiological dataset (SPF); source of D and E curves is teleradiological dataset

At the national level, our teleradiological dataset was composed of 100,018 CT-scans performed during the 36 weeks of the study period. Among those, 19,133 (19.1%) were carried out in the COVID-19 workflow. Table 1 shows how these examinations were distributed across the 10 mainland regions and the 65 partner emergency centres. The epidemiological datasets included 227,677 hospitalisations, 35,970 ICU admissions and 35,993 deaths in hospital. Figure 2 depicts and aligns the epidemiological and teleradiological time series, highlighting a similar shape for all of them, with two spikes corresponding to the first and the second French waves (in March–April and October–November 2020, respectively) separated by an inter-wave period centred on July 2020.

**Table 1:** Characteristics of the teleradiological cohort per region

Regions	No. of CT-scans in COVID-19 workflow^a^	No. of partner emergency centres^b^
Auvergne-Rhône-Alpes	8241/46,049 (17.9)	29/65 (44.6)
Bourgogne Franche Comté	3808/14,639 (26)	5/65 (7.7)
Bretagne	541/4 659 (11.6)	6/65 (9.2)
Grand Est	726/3744 (19.4)	3/65 (4.6)
Hauts de France	657/3460 (19)	2/65 (3.1)
Ile de France	361/2184 (16.5)	3/65 (4.6)
Nouvelle Aquitaine	1815/9973 (18.2)	9/65 (13.8)
Occitanie	1434/8206 (17.5)	6/65 (9.2)
Provence-Alpes-Côte d'Azur	1550/7194 (21.5)	5/65 (7.7)

*No.* number

^a^Data are number of CT-scans performed in the dedicated COVID-19 workflow divided by the total number of CT-scans performed during the same study period (from 2020–03-23 to 2020–11-29) in the partner emergency centres from this region

^b^In total, 65 metropolitan partner emergency centres were included in the study on November 2020

### Correlations between epidemiological and teleradiological time series

**Fig. 3:**
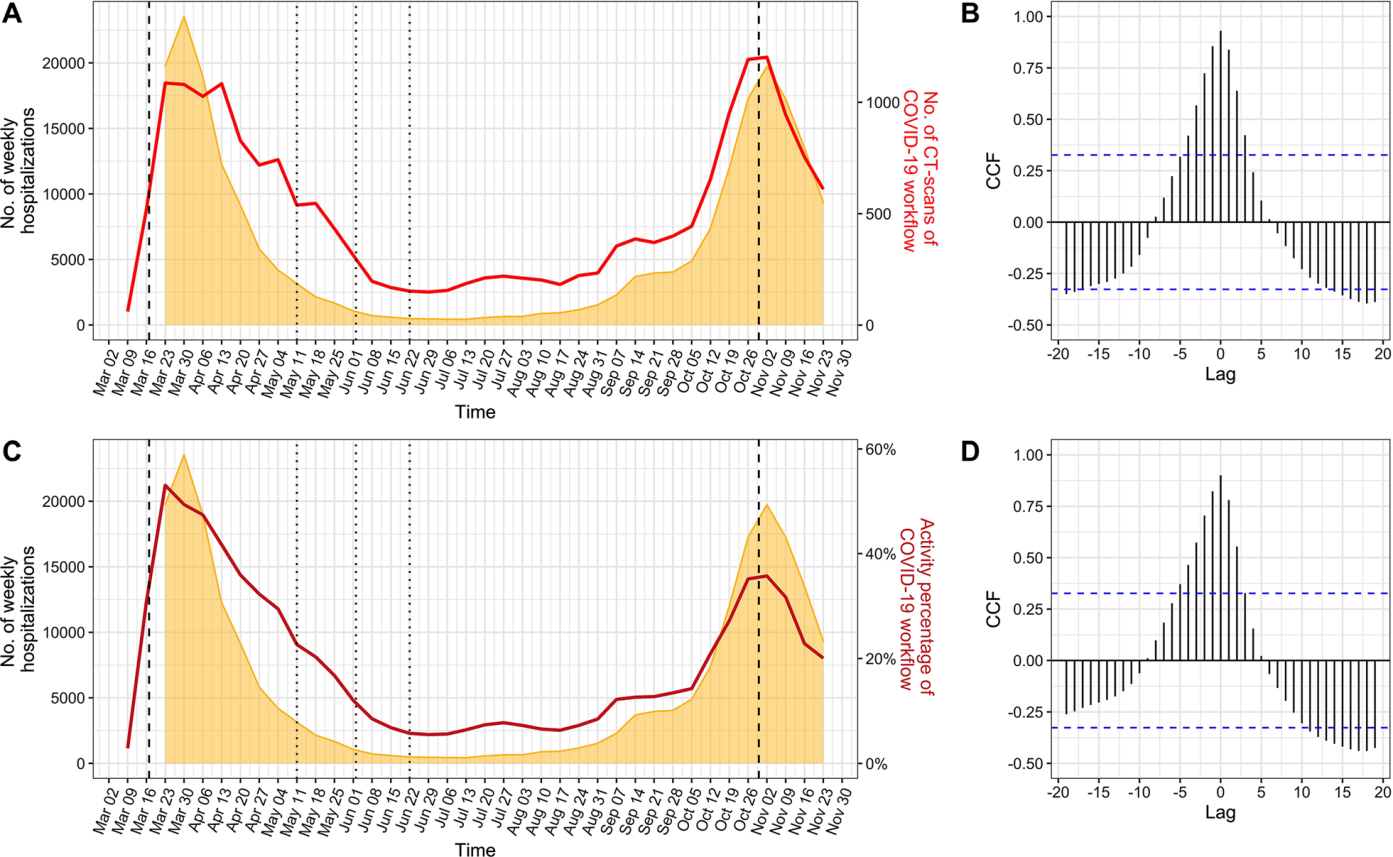
Correlations between the weekly emergency teleradiological time-series and the weekly number of hospitalisations time series. (**a**) Superimposition of the number of hospitalisations with the number of CT-scans performed in the COVID-19 workflow and (**b**) corresponding cross-correlation plot. (**c**) Superimposition of the number of hospitalisations with the percentage of COVID-19-related activity, and (**d**) corresponding cross-correlation plot. A spike above or below the blue lines on the cross-correlation plots indicates a significant correlation of the two time series for the given lag

To assess correlations between the number of hospitalisations and the teleradiological time series on the same week and during prior and past weeks, we applied the CCF. Figure 3 and Table 2 show significant correlations (1) between the weekly number of hospitalisations and the weekly number of CT-scans performed in the COVID-19 workflow from 4 weeks before to 3 weeks after (range of cross-correlation coefficients: 0.42–0.93, the highest being found at lag 0, i.e. the same week) and (2) between the weekly number of hospitalisations and the percentage of activity of the COVID-19 workflow from 5 weeks before to 2 weeks after (range of cross-correlation coefficients: 0.37–0.9, the maximum being also found at lag 0). The coefficients obtained from lag − 2 to lag + 3 were slightly higher with the absolute number of CT-scans than with the percentage of activity. Correlations at higher lags (approximately 15 weeks) corresponded to the similarity between the first and second waves. Details regarding correlations at lag 0 are given in Additional file 1: Data 1.

**Table 2:** Significant consecutive cross-correlations between the weekly emergency teleradiological time series and the weekly number of hospitalisations time series

Lag with no. of hospitalisations	No. of CT-scans performed in the COVID-19 workflow	Percentage of activity of the COVID-19 workflow
5 weeks before	–	0.37
4 weeks before	0.42	0.46
3 weeks before	0.57	0.57
2 weeks before	0.72	0.7
1 week before	0.86	0.82
Same week	**0.93**	**0.9**
1 week after	0.84	0.78
2 weeks after	0.69	0.55
3 weeks after	0.42	–

Data in bold correspond to maximum values

*no.* number

### Identification of primers of the second wave

Table 3 displays the maximal values of each time series during each wave. Regarding the first wave, the maximal number of CT-scans in the COVID-19 workflow and the maximum percentage of activity were reached during the week of 2020-03-23 (1086 CT-scans, 53.1% [1086/2047] activity percentage), while the maximal number of hospitalisations was reached one week later (23,542 patients). Regarding the second wave, the maximal number of hospitalisations, CT-scans and percentage of activity in the COVID-19 workflow were obtained in the week of 2020-11-02 (19,735 patients, 1202 CT-scans and 35.8% [1202/3362], respectively). The maximal percentage of CT-scans compatible with or strongly suggestive of COVID-19 was achieved one week later (74.4%, [702/943]). The ratio between the maximums of the first wave and second wave was higher with the percentage of activity than with the absolute number of CT-scans (0.90 [1086/1202] vs. 1.48 [53.1/35.8]).

**Table 3:** Extrema of the emergency teleradiological time series and the epidemiological time series, during the first wave, inter-wave and second wave, with corresponding dates

Time series	First wave		Inter-waves		Second wave	
	Maximum	Week of maximum	Minimum	Week of minimum	Maximum	Week of maximum
No. of hospitalisations	23,542	2020-03-30	431	2020-07-13	19 735	2020-11-02
No. of ICU hospitalisations	4445	2020-03-30	50	2020-29-06	2 994	2020-11-02
No. of deaths at hospital	3436	2020-04-06	58	2020-08-03	2 875	2020-11-09
No. of CT-scans in the COVID-19 workflow	1086	2020-03-23	148	2020-29-06	1 202	2020-11-02
Percentage of activity of COVID-19 workflow	53.1%	2020-03-23	5.5%	2020-29-06	35.8%	2020-11-02
No. of CT-scans compatible with COVID-19	–	–	24	2020-08-17	793	2020-11-02
Percentage of compatible CT-scans in COVID-19 workflow	–	–	12.4%	2020-08-10	74.4%	2020-11-09
Percentage of compatible CT-scans over all CT-scans	–	–	0.8%	2020-08-10	23.6%	2020-11-09

Lines 1–3: epidemiological data from the French public health agency (Santé Publique France)

Lines 4–8: teleradiological data

*No.* number, *ICU* intensive care unit

**Fig. 4:**
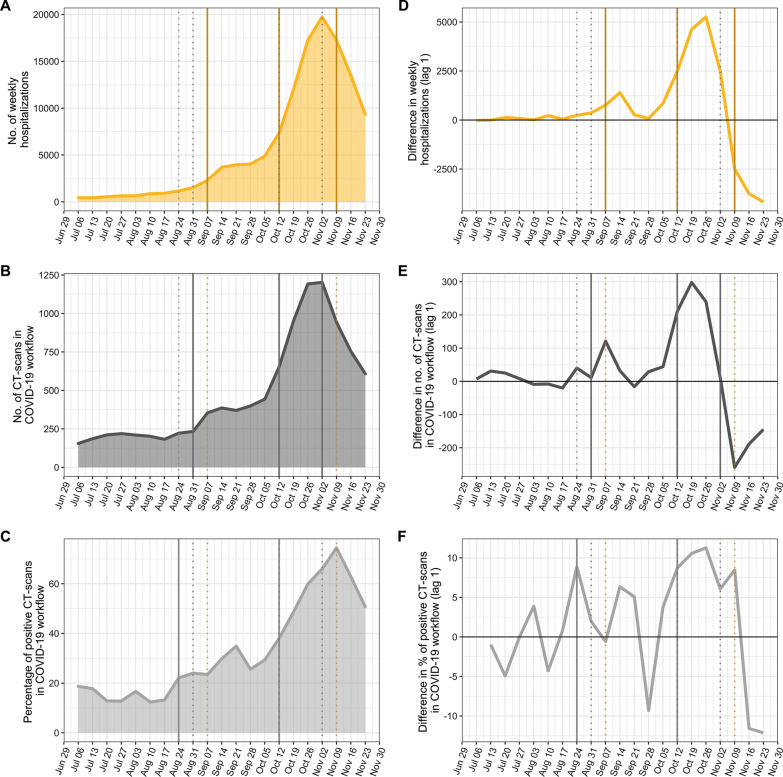
Assessment of breakpoints after the first wave on the following weekly time series: (**a**) no. of hospitalisations, (**b**) no. of CT-scans performed in the COVID-19 workflow by teleradiologists during on-call duty, (**c**) percentage of compatible CT-scans in the COVID-19 workflow. On each plot, the solid vertical lines correspond to the significant breakpoints. The dotted lines correspond to the significant breakpoints for the other time series. (**d–f**) illustrates the lagged difference (*X*(*t*) – X(*t* − 1)) for the three series, respectively, highlighting a spike between the weeks of 2020-08-24 and 2020-09-14

Figure 4 shows the significant breakpoints and structural changes in the number of hospitalisations, CT-scans performed in the COVID-19 workflow and percentage of COVID-19–compatible/strongly suggestive CT-scans from the complete end of the restrictive measures of the first wave to the end of the study. In the three time series, a first plateau could be seen in the second half of August (Fig. 4a–c), which translated to a spike in the differenced time series (Fig. 4d–e). The corresponding breakpoints were identified in the week of 2020-08-24 with the percentage of positive CT-scans in the COVID-19 workflow, 2020-08-31 with the number of CT-scans in the COVID-19 workflow and 2020-09-07 with the number of hospitalisations. The second breakpoints were all identified on the same week, i.e. 2020-10-12.

### Forecasting number of hospitalisations with teleradiological data

The model that passed the Ljung–Box test with the lowest AICC and lowest MAPE in the test set was based on CT(*t* − 1), CT(*t* − 2), H(*t* − 1) and H(*t* − 2) (*p* = 0.1182, AICC = 509.2 and MAPE_test_ = 5.1) (Table 4). Table 4 also presents the performance of models that were based only on CT(*t*), CT(*t* − 1) and CT(*t* − 2), highlighting large errors when considering only the number of CT-scans in the COVID-19 workflow two weeks before to predict the number of hospitalisations in a given week (MAPE_test_ = 127.1), while the other models showed similar intermediate performance (MAPE_test_ = 20 and 20.7, respectively).

**Table 4:** Final predictive models

Model	Equation	MAPE in train set	Ljung–Box Test	MAPE in test set
CT(*t*)	$H\left( t \right) = ~ - {\boldsymbol{3315.30}} + {\boldsymbol{17.03}} \times ~{\boldsymbol{CT}}\left( {\boldsymbol{t}} \right) + {\boldsymbol{196.63}} \times {\boldsymbol{Ld}}\left( {\boldsymbol{t}} \right) + ~\varepsilon \left( t \right) + ~0.84 \times \varepsilon \left( {t - 1} \right)$, with $~\varepsilon \left( t \right)~ \sim {\mathcal{N}}\left( {0,~3420285} \right)$	6.82	0.0490*	20.02
CT(*t* − 1)	$H\left( t \right) = {\boldsymbol{7.05}} \times {\boldsymbol{CT}}\left( {\boldsymbol{t}} - {\boldsymbol{1}} \right) + {\boldsymbol{889.49}} \times {\boldsymbol{Ld}}\left( {\boldsymbol{t}} \right) + \varepsilon \left( t \right) + ~2.27 \times \eta \left( {t - 1} \right) - ~1.93 \times \eta \left( {t - 2} \right) + 0.59 \times ~\eta \left( {t - 3} \right)$, with $~\varepsilon \left( t \right)~ \sim {\mathcal{N}}\left( {0,~924814} \right)$	25.82	0.0387*	20.72
CT(*t* − 2)	$H\left( t \right) = {\boldsymbol{29227}}.{\boldsymbol{22}} - {\boldsymbol{2.68}} \times \user2{CT}\left( {{\boldsymbol{t}} -{\boldsymbol{ 2}}} \right) - {\boldsymbol{1024.94}} \times {\boldsymbol{Ld}}\left({\boldsymbol{t}} \right) + ~\varepsilon \left( t \right) + ~1.95 \times \eta \left( {t - 1} \right) - ~0.96 \times \eta \left( {t - 2} \right)$, with $~\varepsilon \left( t \right)~ \sim{\mathcal{N}}\left( {0,~968004} \right)$	30.85	0.2406	127.13
CT(*t* − 1), CT(*t* − 2), *H*(*t* − 1), *H*(*t* − 2)	$H\left( t \right) = {\boldsymbol{8.60}} \times {\boldsymbol{CT}}\left( {{\boldsymbol{t}} - {\boldsymbol{1}}} \right) -{\boldsymbol{ 4.27}} \times {\boldsymbol{CT}}\left( {\boldsymbol{t}} -{\boldsymbol{ 2}} \right) + {\boldsymbol{149.89}} \times {\boldsymbol{Ld}}\left( {\boldsymbol{t}} \right) + {\boldsymbol{0.97}} \times {\boldsymbol{H}}\left( {{\boldsymbol{t}} -{\boldsymbol{1}}} \right) - {\boldsymbol{0.42}} \times {\boldsymbol{H}}\left( {\boldsymbol{t}} -{\boldsymbol{ 2}} \right) + ~\varepsilon \left( t \right) + ~1.83 \times \eta \left( {t - 1} \right)$, with $~\varepsilon \left( t \right)~ \sim{\mathcal{N}}\left( {0,~479558} \right)$	24.40	0.1182	5.09

The ‘model’ column gives the predictors entered in the algorithm to predict the number of hospitalisations for the week ‘*t*’. Hence, ‘*t* − 1’ and ‘*t* − 2’ are one and two weeks before (i.e. lag − 1 and lag − 2)

The terms in bold correspond to the regression part of the model, and the other terms to the error *η*(*t*) which can be expressed with an auto-regressive integrated moving average (ARIMA) model with *ε*(*t*) an uncorrelated error term (i.e. white noise) following a normal law *N* with variance in parentheses

CT(*x*), where *x* in {t, *t* − 1, *t* − 2}, corresponds to the number of CT-scans performed in the COVID-19 teleradiological emergency workflow during the week ‘*x*’

*H*(*x′*), where *x*′ in {*t* − 1, *t* − 2}, corresponds to the number of patients hospitalised in mainland French hospitals during the week ‘*x*′’

Ld(*t*) is a binary variable that takes the value 1 if France is under national lockdown and 0 otherwise

*ARIMA* auto-regressive integrative moving average, *MAPE* mean absolute percentage error

^*^p < 0.05

**Fig. 5:**
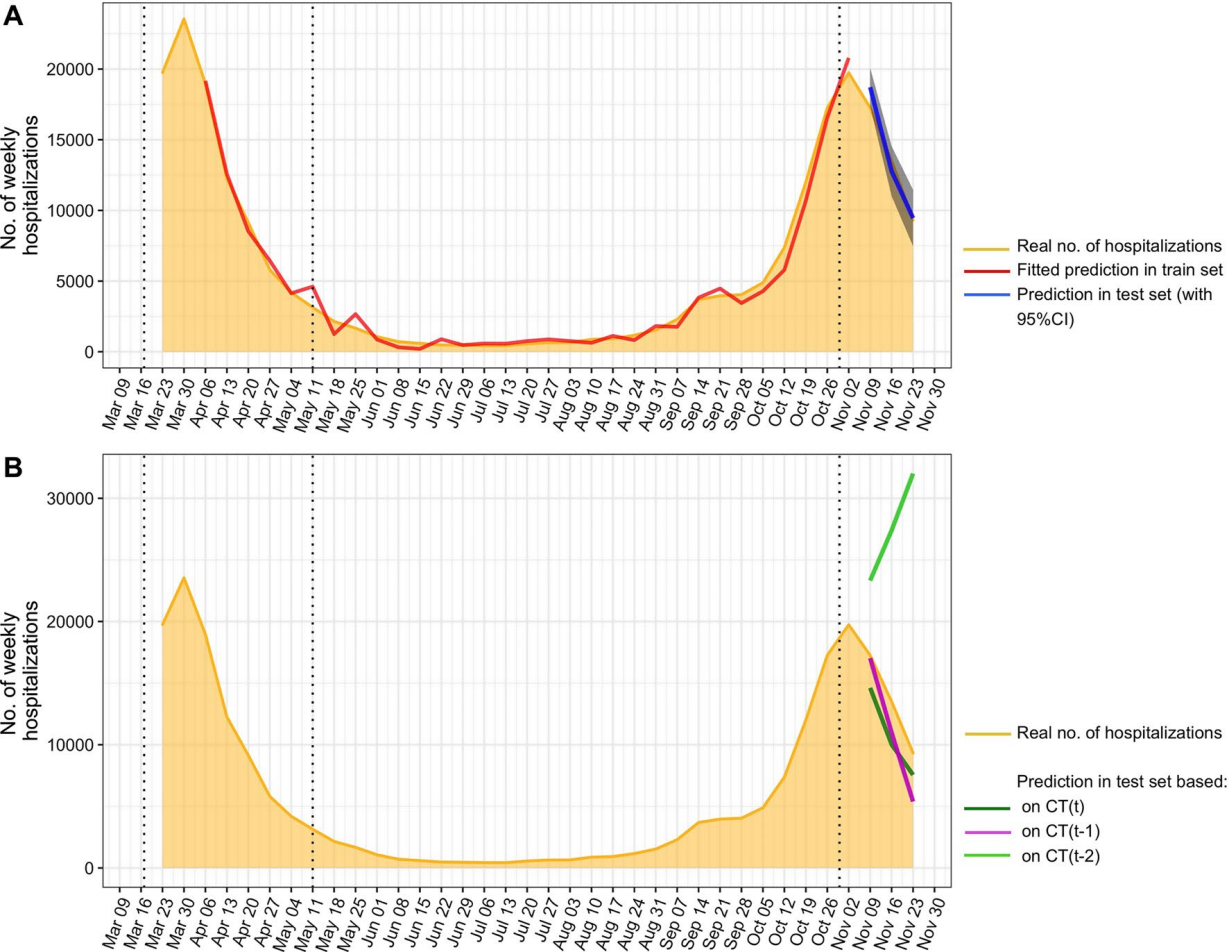
Predictions of the best model (**a**) and the models based on the number of CT-scans performed the same week (CT(*t*)), one week before (CT(*t* − 1)) and two weeks before (CT(*t* − 2)) in the COVID-19 workflow (**b**). The dotted lines correspond to the beginning and the end of the 1^st^ national lockdown and the beginning of the 2nd national lockdown, respectively. Abbreviations: 95%CI: 95% confidence interval, no.: number

The details related to the fitting and performance of the models based on the different combinations of explanatory variables to predict *H*(*t*) are given in Additional file 1: Data 2. Figure 5 shows the superimposition of the fitting and forecasting of the best model, as well as the models based on the number of CT-scans in the COVID-19 workflow alone.

### Time series relationships at the regional scale

**Fig. 6:**
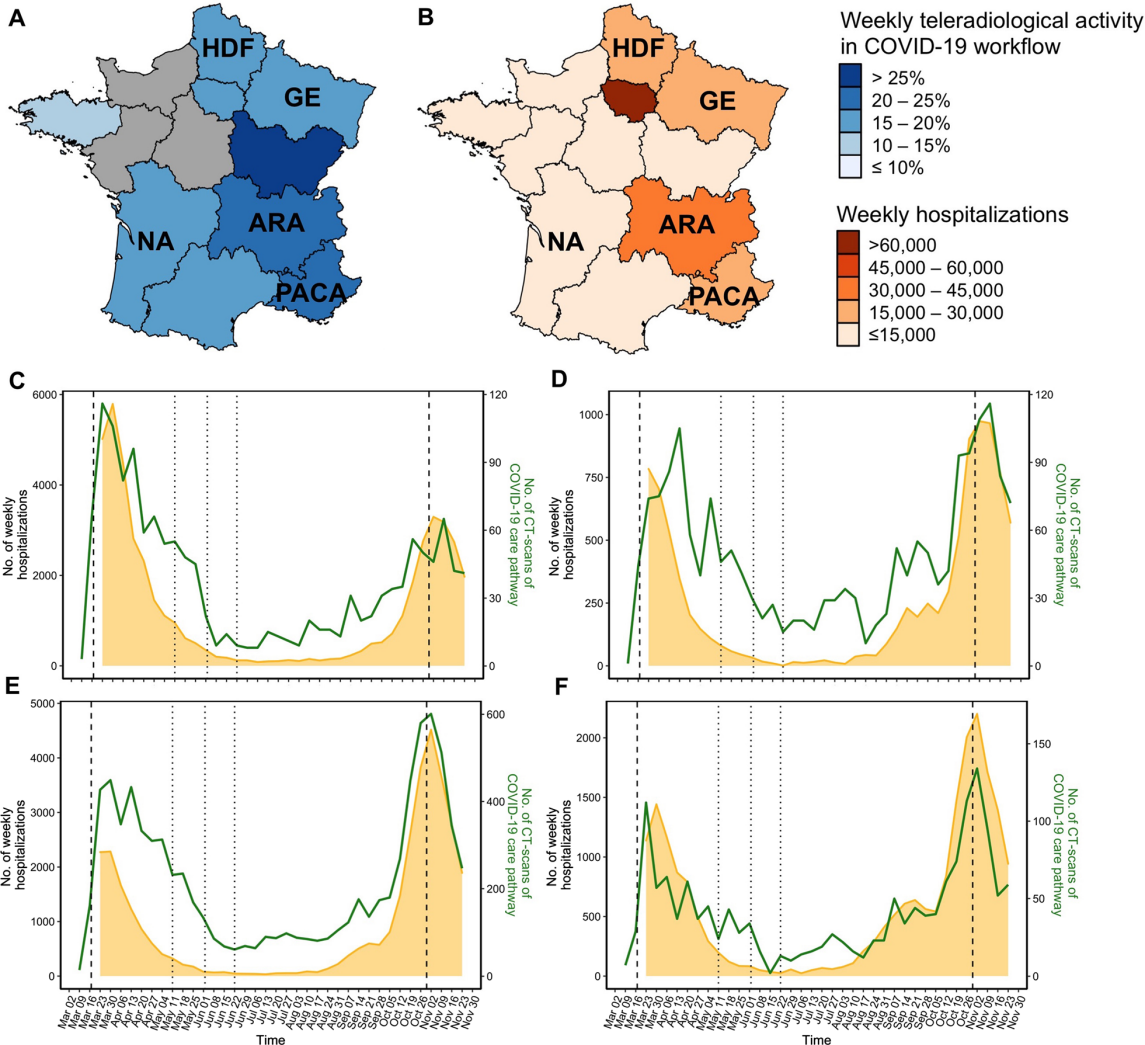
Regional correlations between the teleradiological emergency time-series and number of hospitalisations. French regions with colour-encoding according to the percentage of teleradiological emergency activity in the COVID-19 workflow (**a**) and the number of hospitalisations (**b**) from 2020-03-23 to 2020-11-30. Superimposition of the two time series over the study period in (**c**) the Hauts-de-France (HDF) and Grand-Est (GE) regions; (**d**) the Nouvelle Aquitaine (NA) region; (**e**) Auvergne-Rhône-Alpes (ARA) region and (**f**) the Provence-Alpes-Côte d’Azur region

We focused on four French areas (Nouvelle-Aquitaine [southwest], Hauts-de-France and Grand-Est [northeast], Provence-Alpes-Côte d’Azur [southeast] and Auvergne-Rhône-Alpes [central east]) to investigate whether emergency teleradiology indicators could also be used to help monitor the pandemic at the regional level (Fig. 6). The extremes and their corresponding dates for both waves, as well as the low values in the inter-wave period and the intermediate plateau at the end of summer, were superimposed in the four cases.

## Discussion

This nationwide multicentric prospective study shows that real-time prospective and structured radiological data, possible in our teleradiology structure dedicated to emergency centres, can provide relevant real-time predictive markers of the evolution of COVID-19 pandemic on a national scale. Indeed, our results, gathered since the beginning of the outbreak in France, highlight the strong correlations between the number of hospitalisations per week and the number of CT-scans related to COVID-19 performed in our partner hospitals, especially in the two preceding weeks. These complementary early predictive markers could be used by public health agencies to instantly confirm tendencies or outbreak resurgence or even as new predictors in forecasting models. Furthermore, our findings could translate to smaller geographical levels and help monitor the pandemic over mainland territories.

Despite the growing interest in imaging data integration into population health management [17], the value of nationwide imaging data to monitor and predict the evolution of COVID-19 pandemic through time series analysis has never been demonstrated. Imaging is frequently the gateway to patients’ diagnosis and treatment, and such an approach makes sense. Unfortunately, this approach has been undermined by several factors: heterogeneous practices, lack of compatibility of information and technology tools, time, or free exchange platforms for medical specialists. The rare time series analyses involving imaging mostly depicted trends in general or emergency radiological activity and sometimes developed forecasting models [18–20]. Conversely, analysis of the radiological activity during COVID-19 pandemic has generally consisted of examining changes compared to usual general or emergency activity, but the predictive value of these changes in terms of public health has been poorly investigated, with cohorts of fewer than 5000 patients [21–23].

It should be noted that additional correlations could have been investigated with our cohort, for instance, with the number of ICU admissions, deaths or discharges to home, all provided by *data.gouv.fr*. However, as the chronological succession from hospitalization to ICU admission to death is well described, we purposely chose to focus on the first interaction between COVID-19 patients and the hospital system, namely conventional hospitalisations. Second, we decided to use the absolute number of CT-scans in the COVID-19 workflow instead of the percentage of activity of this workflow because the superimposition of the teleradiological and epidemiological time series during the second wave was less pronounced than during the first wave. We believe that this result was due to the preservation of non-COVID-19 activity during this period, which probably led to lower cross-correlation coefficients from lag − 2 to lag + 2 with the percentage of activity than the absolute values. Furthermore, the weekly number of COVID-19–compatible or strongly suggestive CT-scans was not prospectively collected before the beginning of the inter-wave period, which is why we noted its potential as a sensitive primer for a second wave but not for the elaboration of forecasting models due to too many missing data. Indeed, it is worth noting that this teleradiological variable underwent a significant structural change two weeks before the number of hospitalisations.

The centralised organisation of our teleradiology structure has favoured the implementation of early standardised COVID-19 practices. This COVID-19 workflow label has enabled our support teams to constantly monitor teleradiology activity since the week of 2020-03-09 (i.e. one week before the open-source publication of COVID-19 numbers by SPF), with the main initial intent to rapidly adjust our medical and material resources in this unprecedented context. Eventually, this teleradiological variable could also be used to (1) adapt emergency staff resources, (2) evaluate and adapt lockdown measures at national or regional scales, (3) organise inter-town/state patient transfers and (4) reduce global morbidity and mortality.

For example, our statistics were transmitted weekly to the Regional Health Agencies of Auvergne-Rhône-Alpes and Nouvelle Aquitaine upon their request.

Our study has limitations. First, although more than 100,018 CT-scans performed in our partner hospitals were screened for this study, the regional distribution of activity was not homogeneous, leading to marked noise in some regions in the sub-analysis. In addition, the distribution of activity was not perfectly superimposed with COVID-19 spread in France. For instance, our structure has only three emergency partners in the Ile de France (or Parisian) region, though it was the most seriously impacted (70,217 hospitalisations versus 361 CT-scans performed in the COVID-19 workflow among a total of 2184 CT-scans [16.5%]). Second, we did not include overseas departments because the dynamics of the outbreak did not follow the same curve as in mainland due to geographical isolation. Third, other forecasting algorithms, accuracy measurements and non-radiological predictors (based on meteorological, governmental, behavioural or social data, for example) could improve the model’s performance. However, our demonstration used open-source epidemiological data and a robust and classical algorithm with distinct training and validation sets. Fourth, we did not prospectively collect for which precise reason a CT-scan was required in the emergency services (for instance, initial diagnosis, assessment of the disease extent or worsening in patients already diagnosed as COVID-19 positive). Though all the CT-scans performed for these reasons led to an increase in the global emergency radiological activity due to COVID-19, deepening our assessment with complementary variables (such as: numbers of CT-scans performed for initial diagnosis, for assessment of the disease extent in COVID-19 positive patients and for clinical worsening in COVID-19 positive patients) could have provided more powerful estimators. However, it would have required additional work from the emergency physicians and possible difficulties to choose between the three options.

Fifth, although in general patients come only once at the emergency service for less than 48 h before either returning home or being hospitalised in other departments, we cannot exclude that some patients had more than one chest CT-scan in the COVID-19 workflow. Due to the anonymisation of the full cohort, we were not able to compute the exact number of patients with multiple scans. However, based on another study from our group in which 5 out 938 CT-scans (≈ 0.5%) were performed in a same patient during their visit to the emergency for COVID-19, we could estimate, by extrapolating this ratio, that ≈ 100 patients had two CT-scans in our teleradiological dataset [24]. Consequently, comparing this number with the total number of CT-scans (i.e. 100,018) and the total number of CT-scans in the COVID-19 workflow (i.e. 19,133), we do not believe that it could have significantly biased our findings. Sixth, as previously explained, we chose not to use the number of chest CT-scan with a positive report (i.e. with findings strongly suggestive/compatible with the COVID-19 diagnosis) in the forecasting models because these data were only prospectively collected since the French inter-wave period. We would have had to exclude all the observations from the first wave. However, we believe that using the French Society of Radiology scoring system could provide more powerful predictors in future radiological forecasting models—although strongly suggestive/compatible conclusions with COVID-19 diagnosis do not systematically mean positive RT-PCR despite the excellent accuracy of the French Society of Radiology scoring system [4]. Finally, it is important to consider that existing indicators based on hospitalisations should become less accurate with vaccination, necessitating original indicators. Teleradiology variables could be valuable given that (1) emergency imaging will always remain a gateway to patients’ diagnosis and (2) our structured organisation allows real-time analysis of COVID-19 workflow data.

## Conclusion

In conclusion, this prospective nationwide study demonstrates that structured radiological networks enable the rapid aggregation of early relevant indicators of COVID-19 pandemic on a national scale. Our results suggest that scrutinising the dynamics of radiological activity in emergencies could provide early and accurate estimation of the number of hospitalisations in the same period at the national level but also predict the short-term evolution of the pandemic. This approach paves the way for original real-time complementary indicators for public health agencies to improve public health models and monitor preventive measures, as well as adjust human resources. Thus, our findings illustrate the potential value of integrating radiological data to support public health management and stress the need for collaborative nationwide and international radiological networks and platforms.

## Supplementary Information

**Additional file 1.** Supplementary data.

## Data Availability

Epidemiological dataset is available on Santé Publique France website. Radiological dataset can be requested to the corresponding author.

## Abbreviations

95% CI: 95% Confidence interval
AICC: Akaike information criterion corrected
ARIMA: Auto-regressive integrated moving average
CCF: Cross-correlation function
COVID-19: Coronavirus disease 2019
CT(*t*): Number of CT-scans performed in the COVID-19 workflow in a given week ‘*t*’
*H*(*t*): Number of hospitalisations in a given week ‘*t*’
ICU: Intensive care unit
MAPE: Mean absolute percentage error
SPF: *Santé Publique France* (French Public Health Agency
