# Supplementary material for: Emergency teleradiological activity is an epidemiological estimator and predictor of the covid-19 pandemic in mainland France
Source: Insights Imaging. 2021 Jul 22;12:103. doi: 10.1186/s13244-021-01040-3 (PMC8295630; doi:10.1186/s13244-021-01040-3)
Supplement: Supplementary file 1 — Additional file 1. Supplementary data. [file 13244_2021_1040_MOESM1_ESM.docx]

**Additional file 1: Data**

**SUPPLEMENTARY DATA 1a.** Scatter plots between the teleradiological emergency variables (i.e. number of CT-scans performed in the COVID-19 workflow each week and corresponding percentage of activity of this workflow relative to the total number of CT-scans performed during the on-call duty) and the epidemiological variables. Each point corresponds to a week (from the week beginning the 2020-03-23 to the week finishing on 2020-11-29 (included) (n = 36 weeks). The weeks are connected by an arrow.


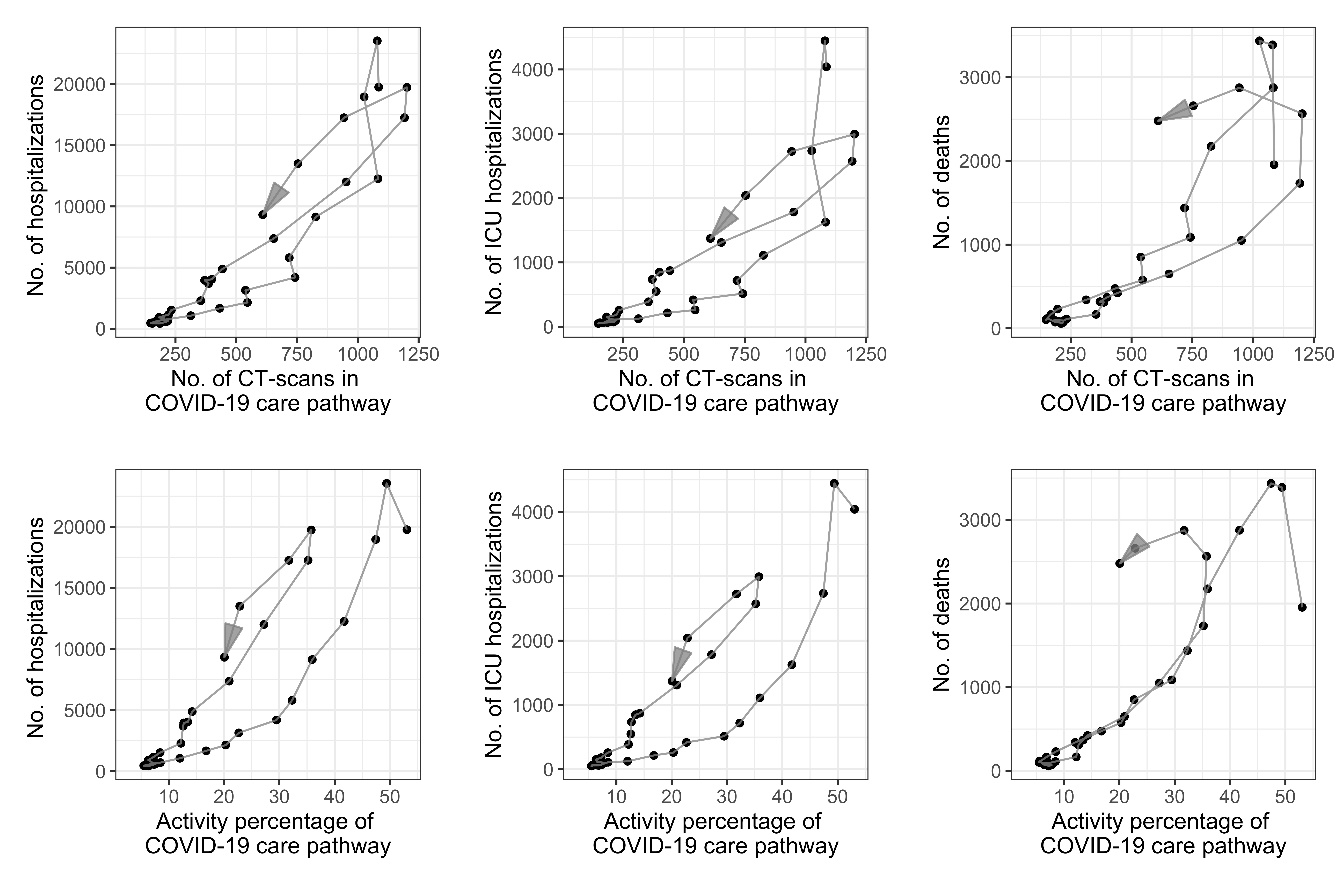


**SUPPLEMENTARY DATA 1b.** Assessment of the correlations between non-lagged teleradiological and epidemiological variables, with the Kendall test**.**

| **Teleradiological variables** | **Epidemiological variables** | **Kendall tau** | **p_value** |
| --- | --- | --- | --- |
| No. CT-scans in COVID-19 workflow | No. of weekly hospitalizations | 0.813 | <0.0001*** |
| No. CT-scans in COVID-19 workflow | No. of weekly admissions in ICU | 0.8 | <0.0001*** |
| No. CT-scans in COVID-19 workflow | No. of deaths | 0.708 | <0.0001*** |
| Percentage of activity of COVID-19 workflow | No. of weekly hospitalizations | 0.79 | <0.0001*** |
| Percentage of activity of COVID-19 workflow | No. of weekly admissions in ICU | 0.759 | <0.0001*** |
| Percentage of activity of COVID-19 workflow | No. of deaths | 0.762 | <0.0001*** |

NOTE. Abbreviations: ICU: Intensive care unit, No.: number.

***: p<0.001

**SUPPLEMENTARY DATA 2.** Summary of the performance of all the models in the train and test cohorts.

| **Models** | **Train** | | | **Test** | **Model error** |
| --- | --- | --- | --- | --- | --- |
|  | **AICC** | **MAPE** | **Ljung-Box test p-value** | **MAPE** |  |
| CT(t) + Ld | 599.3 | 6.8 | 0.0490* | 20. | ARIMA(0,0,1) |
| CT(t-1) + Ld | 548. | 25.8 | 0.0387* | 20.7 | ARIMA(3,0,0) |
| CT(t-2) + Ld | 537.1 | 30.9 | **0.2406** | 127.1 | ARIMA(2,0,0) |
| CT(t) + CT(t-1) + Ld | 546.7 | 30.7 | 0.0076* | 8.2 | ARIMA(2,0,0) |
| CT(t-1) + CT(t-2) + Ld | 526 | 28.9 | **0.0853** | 44.1 | ARIMA(2,0,2) |
| CT(t) + H(t-1) + Ld | 568.1 | 29.7 | 0.0002*** | 8.6 | ARIMA(2,0,2) |
| CT(t-1) + H(t-1) + Ld | 545.6 | 28.8 | **0.0839** | 27.7 | ARIMA(3,0,0) |
| CT(t-2) + H(t-2) + Ld | 531.7 | 30. | 0.0420* | 133.3 | ARIMA(2,0,0) |
| CT(t) + CT(t-1) + CT(t-2) + Ld | 523.1 | 23.4 | 0.0003*** | 15.7 | ARIMA(2,0,2) |
| CT(t) + CT(t-1) + H(t-1) + Ld | 570.8 | 24.2 | **0.1847** | 20. | ARIMA(1,0,0) |
| CT(t-1) + CT(t-2) + H(t-1) + Ld | 520.5 | 21.1 | **0.2114** | 26. | ARIMA(1,0,0) |
| CT(t) + CT(t-1) + CT(t-2) + H(t-1) + Ld | 251.9 | 43.3 | **0.1212** | 19.9 | ARIMA(0,0,0) |
| CT(t) + CT(t-1) + H(t-1) + H(t-2) + Ld | 507.1 | 21.9 | 0.0228 | 4. | ARIMA(1,0,0) |
| CT(t-1) + CT(t-2) + H(t-1) + H(t-2) + Ld | 509.2 | 24.4 | **0.1182** | 5.1 | ARIMA(1,0,0) |
| CT(t) + CT(t-1) + CT(t-2) + H(t-1) + H(t-2) + Ld | 497.3 | 23.5 | 0.0149* | 20.1 | ARIMA(0,0,1) |

NOTE. The ‘models’ column gives the predictor entered in the algorithm to predict the number of hospitalizations for the week ‘t’. Hence, ‘t-1’ and ‘t-2’ are one and two weeks before.

CT(x), where x in {t, t-1, t-2}, corresponds to the number of CT-scans performed in the COVID-19 teleradiological emergency workflow during the week ‘x’.

H(x’), where x’ in {t-1, t-2}, corresponds to the number of patients hospitalized in mainland French hospitals during the week ‘x’’.

Ld is a binary variable that takes the value 1 if France is under national lockdown and 0 otherwise.

Other abbreviations: AICC: Akaike information criterion corrected, ARIMA: auto-regressive integrated moving average, MAPE: mean absolute percentage error.

The model highlighted in blue corresponds to the best model compromise in term of AICC in train set, MAPE in train and validation sets and no anomaly with the residuals.

*: p<0.05, **: p<0.005, ***: p<0.001
